# Case report: Dramatic impact of DNA next generation sequencing results using specific targeted therapies—*ALK* and *PIK3CA*

**DOI:** 10.3389/fonc.2024.1462930

**Published:** 2024-11-26

**Authors:** Stav Cullum, Hlu Vang, Michael Glover, Howra Alammarah, Heather Morton, Nancy Pham, Mobeen Rahman, Saad A. Khan

**Affiliations:** ^1^ Division of Oncology, Department of Medicine, Stanford Cancer Institute and Stanford University, Stanford, CA, United States; ^2^ Department of Otolaryngology/Head & Neck Surgery Divisions, Stanford University, Stanford, CA, United States; ^3^ Department of Radiology, Stanford University, Stanford, CA, United States; ^4^ Department of Pathology, Stanford University, Stanford, CA, United States

**Keywords:** ALK inhibitor, PI3 kinase inhibitor, DNA NGS, RNA NGS, precision oncology

## Abstract

In the era of targeted therapies, the clinical importance and utility of next-generation sequencing (NGS) has expanded significantly. Owing to the relative ease and financial feasibility of NGS, the use of personalized treatment strategies has the potential to revolutionize cancer care. In this case report, we explored the use of NGS in salivary gland carcinoma (SGC) and spindle cell neoplasm of the scalp. In our patient with SGC, NGS revealed a GPHN-ALK variant that allowed off-label treatment with alectinib, with a remarkable response in primary and metastatic foci. Similarly, the use of NGS in a cutaneous neoplasm in which no definitive diagnosis could be reached by pathology and which had progressed through standard of care treatment elucidated a PIK3CA mutation in which alpelisib was added and ultimately halted POD. Here, we discuss the use of NGS, future projections, and our recommendations.

## Introduction

In the age of targeted therapies, the value and applicability of next-generation sequencing (NGS) in clinical settings has grown exponentially. NGS is technically easy to perform on patient tumor specimens and is financially feasible for tertiary medical cancers. Therefore, personalized treatment strategies have the potential to revolutionize cancer care (8).

Genomic abnormalities that respond well to specific targeted therapies are limited to cancers that arise in specific organs. Examples include erlotinib, which was Food and Drug Administration (FDA) approved for use in lung cancer with Epidermal Growth Factor Receptor (EGFR) mutations. There is increased interest in “tissue-agnostic” drug approvals, where genomic abnormality is an indication for targeted therapy independent of the cancer tissue of origin. In modern oncology clinics, there is a gap in which genomic abnormalities are identified in the DNA and RNA of patients with cancer, but drugs are approved for use in other distinct tumor types. Patients whose tumor DNA contains abnormalities but without a tumor-agnostic FDA-approved drug in that cancer type present a significant challenge for oncologists in making evidence-based recommendations.

Certain gene aberrations may play a significant role in the molecular pathogenesis of salivary gland carcinomas (SGC) (5–7). Androgen receptor (AR), human epidermal growth factor receptor (HER2), NTRK gene fusion, anaplastic lymphoma kinase (ALK) rearrangement, RET fusion, and BRAF V600E have all been implicated in the oncogenesis of SGC (2). Of these, AR and HER2 are the most commonly expressed changes in this cancer type; thus, immunohistochemical staining is often completed for these variants (6). In contrast, the tyrosine kinase receptor ALK is rare in SGC (5). Similarly, BRAF, NRAS, NF1, KIT, GNAQ/GNA11, CDKN2A, and PTEN mutations are common genomic alterations in melanoma; NOTCH, CDKN2A, and TP53 in squamous cell carcinoma and NTRK 1–3 in spindle cell sarcoma; and PI3KCA variants are common in ovarian, breast, and lung cancers, but rare in cutaneous malignancies.

In this case report of two similar patients, we explored the use of next-generation sequencing in an SGC and a spindle cell neoplasm of the scalp that responded exceptionally well to alectinib and alpelisib. The durable complete response in these cases suggests that the identification of targetable mutations may be worthwhile to consider routinely in rare or otherwise complex cases.

## Case presentation

### An androgen receptor–positive salivary gland carcinoma found to have GPHN::ALK and AR-V7 splice variant using institutional NGS testing

**Figure 1:**
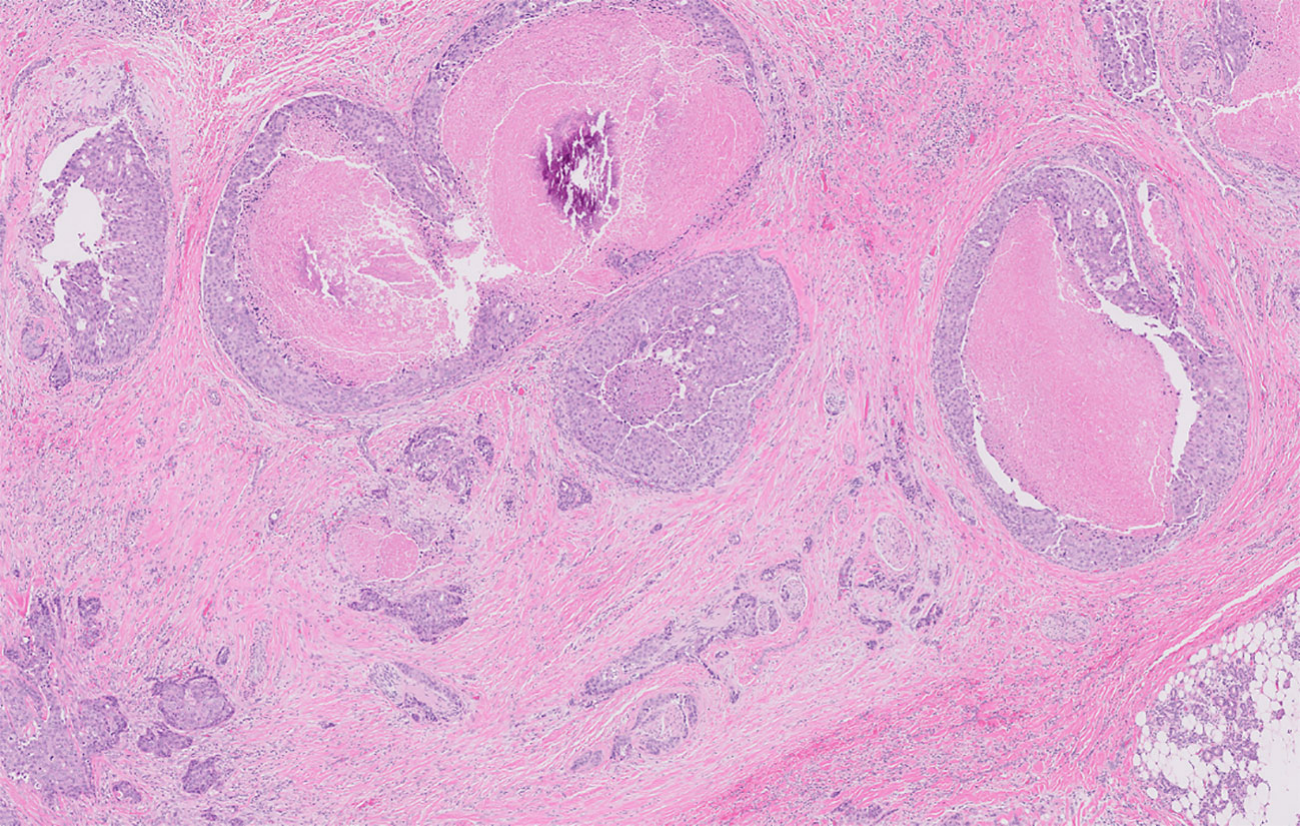
Infiltrative carcinoma with areas of perineural invasion. Areas of *in situ* disease are also characterized by scattered cribriform growth patterns and comedonecrosis.

**Figure 2:**
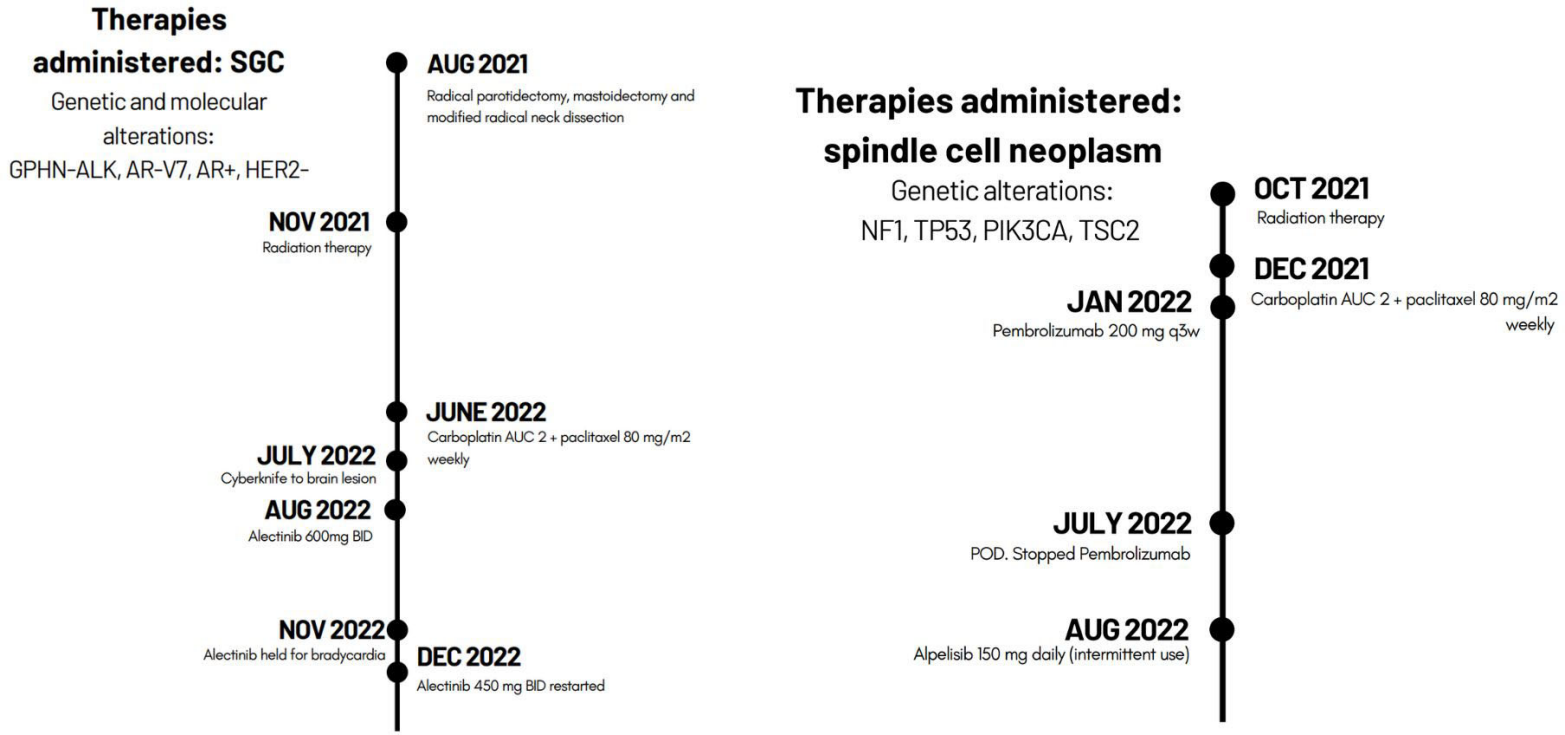
Genetic alterations and timeline of therapies administered to each patient during the course of disease.

**Figure 3:**
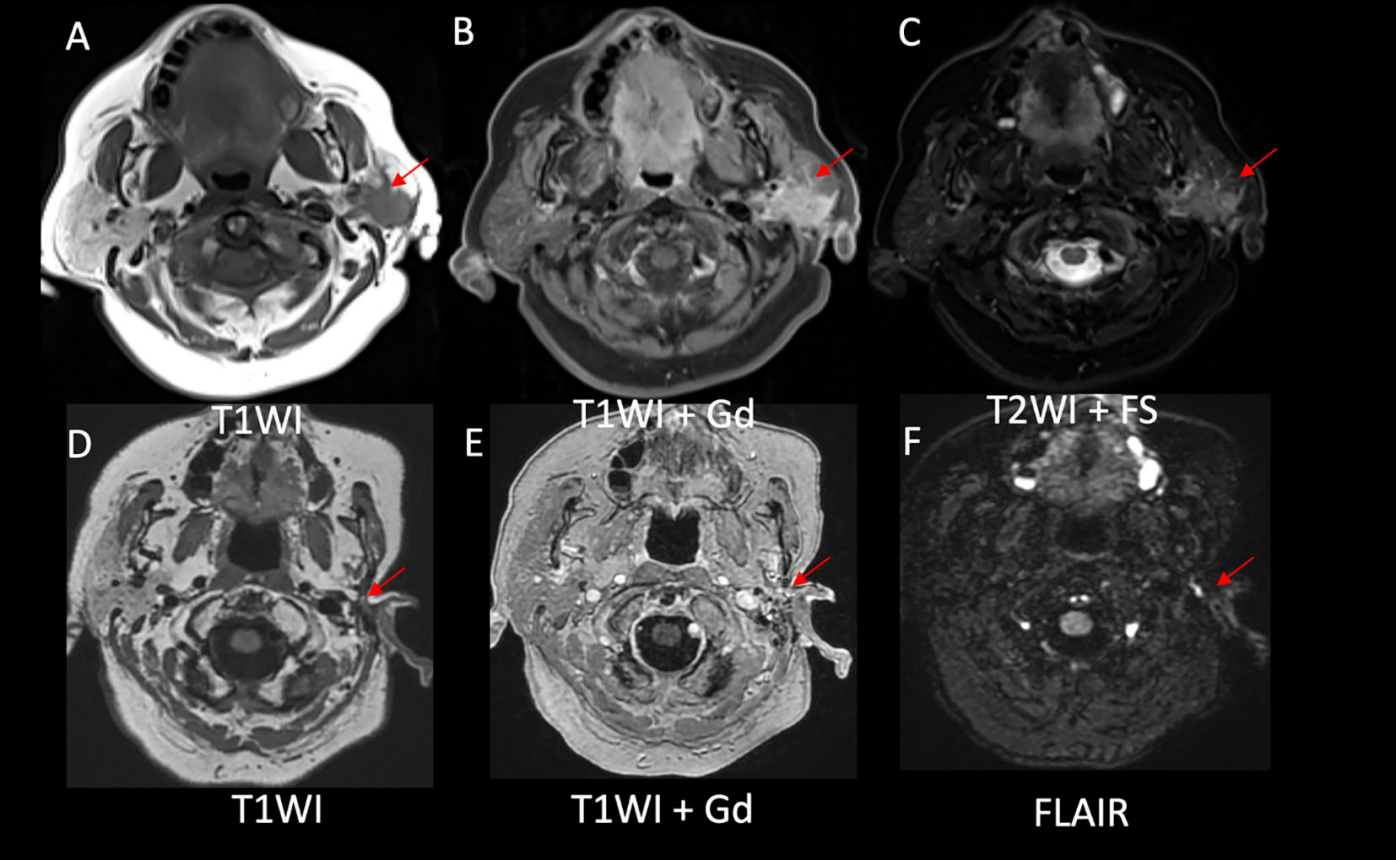
Evidence of a decreased disease burden after alectinib initiation. **(A)** Left parotid mass with ill-defined borders on T1WI, **(B)** homogenous enhancement on postcontrast T1WI, and **(C)** intermediate signal on T2WI in axial images. Postsurgical and posttreatment axial images **(D-F)** showed no evidence of disease in the left parotid bed.

A 74-year-old non-smoker female with no significant medical history presented to our institution with biopsy-proven poorly differentiated pT4a pN3b (AJCC 8th edition) androgen receptor (AR)-positive, HER2-negative metastatic salivary duct carcinoma of the left parotid gland (**Figure 1**). Evidence of metastatic disease included 17/39 lymph nodes in the ipsilateral neck with perineural and lymphovascular invasion as well as pulmonary nodules. The patient received adjuvant radiation (60 Gy/30 Fx) and declined systemic therapy. Postoperative post-radiotherapy imaging showed progression of the pulmonary disease without signs of local recurrence. Pulmonary nodules were biopsied and found to be consistent with SGC. Over the course of the following four months, interval imaging was performed for metastatic intracranial disease with a left cerebellar lesion. Initiation of carboplatin and paclitaxel decreased pulmonary metastases, but the cerebellar lesion remained unchanged. The patient underwent CyberKnife treatment for solitary cerebellar lesions. Finally, off-label treatment with alectinib was initiated after NGS revealed a GPHN::ALK fusion and an AR-V7 splice variant (**Figure 2**). After approximately a month of 600 mg twice daily dosing of alectinib, imaging (1, 2) showed an interval decrease in metastatic burden involving both the lungs and the brain (**Figure 3**). The patient’s case was discussed at our institution’s Head and Neck Tumor Board, and it was recommended to continue alectinib to peak response. Ten months after treatment onset with alectinib, and despite a dose reduction to 450 mg twice daily secondary to ALK inhibitor-associated bradycardia, the patient continues to do well clinically and radiographically.

AR-V7 androgen receptor splice variation is also significant in the treatment of androgen receptor-positive salivary gland cancer (2). Owing to the exceptional response of the patient’s cancer to alectinib, the patient did not receive androgen receptor blockade therapy. This particular variant has been reported to predict an inferior response to androgen blockers (14) and may delay the decision to use androgen blockers in the future.

### A poorly differentiated malignant spindle cell neoplasm showing PIK3CA P124L missense variant on institutional DNA-NGS testing

**Figure 4:**
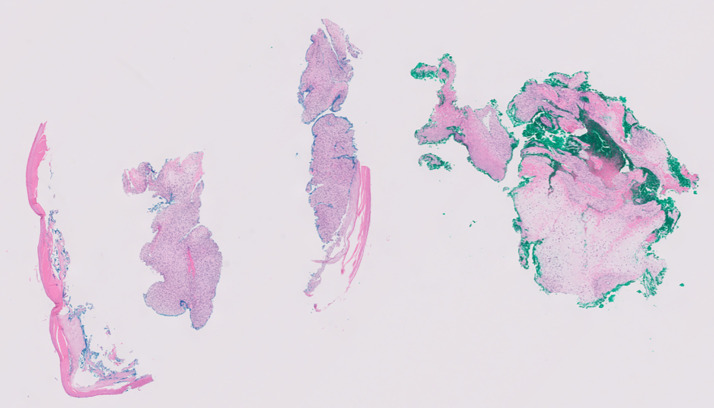
Spindle cell neoplasm with focal cartilaginous matrix formation with higher magnification of spindle cell.

**Figure 5:**
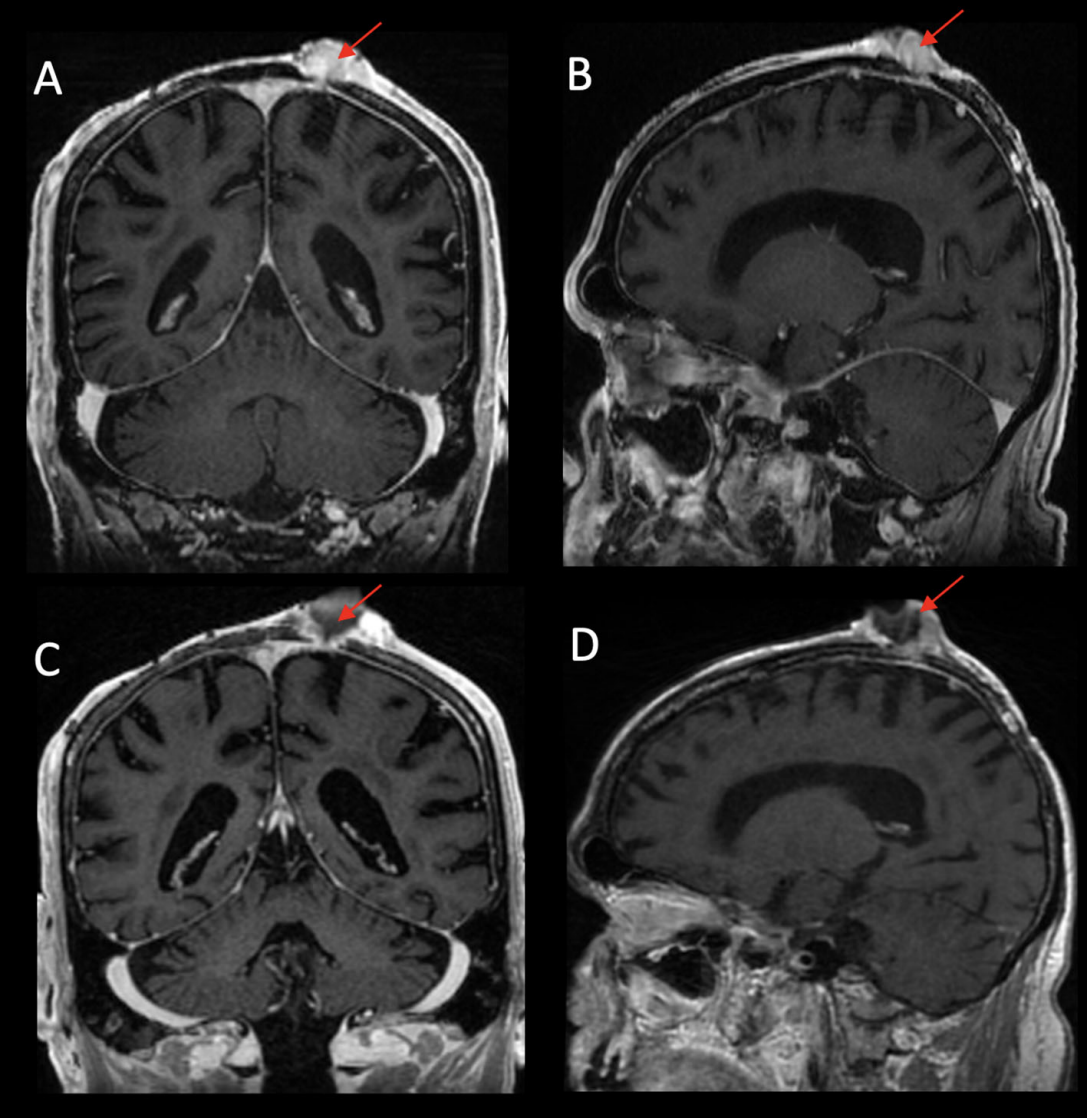
Left parietal scalp exophytic mass with heterogeneous enhancement eroding through the calvarium to involve the underlying dura on post-contrast T1WI coronal **(A)** and T1WI sagittal **(B)** images. Post-treatment images demonstrate interval necrosis of the mass with decreased size of the solid component of the tumor on post-contrast T1WI coronal **(C)** and T1WI sagittal **(D)** images.

An 82-year-old male with a history of biopsy-proven melanoma of the scalp presented to our institution with moderately differentiated invasive squamous cell carcinoma at the posterior skin graft edge of a previously excised melanoma. The patient underwent Mohs surgery and flap reconstruction with adjuvant radiotherapy (RT) plans. However, before radiation could be initiated, the patient presented with a new scalp nodule with histology suggestive of a malignant spindle cell neoplasm (**Figure 4**). Differential diagnosis, as reviewed by our Cutaneous Oncology tumor board, included dedifferentiated melanoma with osseous differentiation, sarcomatoid squamous cell carcinoma, or spindle cell sarcoma; no definitive diagnosis could be made. After the tumor board discussion, radiation to the scalp with or without immunotherapy and institutional DNA-NGS to better characterize the patient’s disease was recommended. RT was initiated. However, due to the slow tumor response to radiation alone, systemic therapy with carboplatin/paclitaxel was administered. Local disease progression was observed one month later. The patient’s case was reviewed by our Head and Neck Tumor Board, with recommendations for the addition of immunotherapy given the rapid disease progression, and pembrolizumab was selected. NGS confirmed the presence of NF1 E163X, NF1 R1830G, TP53 D281N, PIK3CA P124L, and TSC2 Q1115X, as well as a tumor mutation burden of 54 (**Figure 2**). After the initial response to immunotherapy over the course of five months, imaging revealed disease progression. Given the known PIK3CA mutation, the PI3KCA-inhibitor alpelisib, classically used in breast cancer, was recommended for the next course of treatment. The patient was started on aleplisb (150 mg, twice daily). Despite inconsistent use due to side effects, including rash, edema, and fever, after ten months of treatment, the patient has maintained clinical stability as well as tumor shrinkage on most recent imaging (**Figure 5**). Importantly, the use of this targeted therapy has been successful despite intermittent dose reduction and self-discontinuation, with the patient independently adjusting his dose and frequency based on his current experience of side effects.

## Discussion

Salivary gland carcinomas are a heterogeneous group of rare and aggressive carcinomas with high rates of local recurrence and distant metastases, despite curative intent with surgery with or without adjuvant radiation or systemic chemotherapy for metastatic disease. Chemotherapy is typically a platinum-based regimen with targeted therapy that is useful in relevant cases (10, 11). The ALK inhibitor alectinib has not been widely used in SGCs, as only 4%–6% of cases exhibit ALK rearrangement. In the case discussed here, alectinib successfully halted disease progression in a patient with an otherwise refractory tumor that did not respond to the standard of care carboplatin/paclitaxel. Androgen deprivation therapy was of less benefit in this patient, as AR-V7 positivity has been shown to predict a poor prognosis in other forms of cancer and is an independent risk factor for overall survival in certain groups (13). Additionally, alectinib was preferred given its central nervous system (CNS) penetrance and known metastatic sites, including the cerebellum. As a rare genetic variant, identification of this tumor’s ALK alteration was only elucidated via NGS, and ultimately precipitated an exceptional response for this patient.

Similarly, the use of NGS to identify a PIK3CA missense mutation in a patient without a definitive pathological diagnosis of cutaneous malignancy has allowed targeted treatment of an otherwise complex tumor with uncertain histology. The wide differential diagnosis considered in this patient’s case based on tumor histology was accompanied by an equally diverse group of potential systemic treatment options, ranging from AIM (doxorubicin, ifosfamide, mesna) or anthracycline-based regimens for metastatic disease in the case of sarcoma versus platinum-based regimens or immunotherapy in the case of squamous cell carcinoma or melanoma. In this case, instead of selecting the treatment based on the most likely, but not necessarily definitive, diagnosis, given the tumor’s known PIK3CA gain-of-function effect, alpelisib was considered and ultimately found to be extremely successful even with imperfect use by the patient.

As discussed elsewhere, NGS has opened the door to the identification of targeted therapies for uncommon cancers, many of which are treated less specifically and efficaciously (5). While genetic testing and the use of precision medicine are becoming more widespread, the incorporation of these tools into everyday clinical practice is not yet ubiquitous. Furthermore, there is significant room for improvement in the insurance coverage of off-label therapies, which is appropriate based on NGS results. Currently, access to NGS technology and appropriately targeted therapies is inconsistent; insurance coverage often relies on using standard of care or well-studied alternatives, in which rare tumor types with rare targetable mutations are unlikely to be in abundance (5).

In both cases, the patient samples were tested for mRNA expression using the institutional platform Solid Tumor Actionable Mutation Panel for Fusions (Fusion STAMP). The novel *ALK* fusion was identified using an mRNA profiling panel, while the *PIK3CA* mutation was identified only on DNA NGS but not on the Fusion STAMP panel. This is consistent with the technical abilities of sequencing platforms, in which novel fusions may be better identified by RNA next-generation sequencing, while gene mutations can be identified on traditional DNA NGS. Increasingly, patients at tertiary care centers are getting both DNA and RNA sequencing performed routinely, although the exact utility and cost-effectiveness of a universal testing approach are unclear.

Finally, the two outcomes described in our report highlight the benefits of DNA- and RNA-NGS testing with an associated good response to off-label treatment, but they are not universally representative. For patients treated in resource-limited (money and knowledge) environments, access to NGS testing and off-label drug use may not be possible. These represent patients with cancer who may die early due to lack of awareness of or access to potentially efficacious treatment options. Conversely, in well-resourced centers, cancer patients may receive targeted therapies based on NGS results rather than participate in clinical trials, which has the potential to delay the FDA approval of agents that would have been more effective (9, 10). The resources devoted to universal DNA and RNA-NGS testing may be better utilized by creating a database where the results of DNA and RNA-NGS identify genetic abnormalities treated with off-label targeted therapies. Any mechanism that collects all information would likely overcome the bias of only reporting excellent outcomes, but would still not fully capture the potential toxicities experienced by these patients.

To make a potentially efficacious treatment available to patients, sequencing technologies such as NGS and the use of biomarker-specific treatments, even if off-label, should be considered in all patients with refractory, undifferentiated, or otherwise complex disease (3, 4). We agree that with the power of NGS and knowledge of tumor-specific alterations, which treatments are considered should be agnostic of tissue type (5, 12). A thoughtful incorporation of technologies into standard oncology practices, along with recording outcomes (both positive and negative), would likely increase the number of patients experiencing exceptional response results, while also reducing the number of patients who are given futile treatment.

## Data Availability

The raw data supporting the conclusions of this article will be made available by the authors, without undue reservation.
